# Activation of AMPK inhibits cervical cancer cell growth through AKT/FOXO3a/FOXM1 signaling cascade

**DOI:** 10.1186/1471-2407-13-327

**Published:** 2013-07-03

**Authors:** Mingo Ming Ho Yung, David Wai Chan, Vincent Wing Sun Liu, Kwok-Ming Yao, Hextan Yuen-Sheung Ngan

**Affiliations:** 1 Departments of Obstetrics and Gynaecology, LKS Faculty of Medicine, The University of Hong Kong, Hong Kong SAR, People’s Republic of China; 2 Departments of Biochemistry, LKS Faculty of Medicine, The University of Hong Kong, Hong Kong SAR, People’s Republic of China

**Keywords:** AMPK, FOXM1, FOXO3a, Cervical cancer

## Abstract

**Background:**

Although advanced-stage cervical cancer can benefit from current treatments, approximately 30% patients may fail after definitive treatment eventually. Therefore, exploring alternative molecular therapeutic approaches is imperatively needed for this disease. We have recently shown that activation of AMP-activated protein kinase (AMPK), a metabolic sensor, hampers cervical cancer cell growth through blocking the Wnt/β-catenin signaling activity. Here, we report that activated AMPK (p-AMPK) also inhibits cervical cancer cell growth by counteracting FOXM1 function.

**Methods:**

Effect of the activation of AMPK on FOXM1 expression was examined by hypoxia and glucose deprivation, as well as pharmacological AMPK activators such as A23187, AICAR and metformin. RT Q-PCR and Western blot analysis were employed to investigate the activities of AMPK, FOXM1 and AKT/FOXO3a signaling.

**Results:**

Consistent with our previous findings, the activation of AMPK by either AMPK activators such as AICAR, A23187, metformin, glucose deprivation or hypoxia significantly inhibited the cervical cancer cell growth. Importantly, we found that activated AMPK activity was concomitantly associated with the reduction of both the mRNA and protein levels of FOXM1. Mechanistically, we showed that activated AMPK was able to reduce AKT mediated phosphorylation of p-FOXO3a (Ser253). Interestingly, activated AMPK could not cause any significant changes in FOXM1 in cervical cancer cells in which endogenous FOXO3a levels were knocked down using siRNAs, suggesting that FOXO3a is involved in the suppression of FOXM1.

**Conclusion:**

Taken together, our results suggest the activated AMPK impedes cervical cancer cell growth through reducing the expression of FOXM1.

## Background

Cervical cancer results from uncontrolled growth of malignant cells started within the uterine cervix and is one of the most common malignancies in women worldwide [1-3]. Although this disease is almost preventable with routine genetic screening and vaccination, more than 80% of cervical cancers with a majority in the advanced stage are currently found in developing countries including China, leading to a high risk of recurrence and poor survival [2,4]. Thus, there is a compelling need to explore novel therapeutic interventions for this disease.

Emerging evidence suggests that targeting cancer cell metabolism is a promising therapeutic approach in human cancers. AMP-activated protein kinase (AMPK) is a known cellular metabolic sensor and plays an important role in the control of energy homeostasis in response to external stresses [5-8]. Recent studies have documented that pharmacological activation of AMPK is able to block cancer cell growth in various human cancers [8-11]. Indeed, we have previously reported that pharmaceutical AMPK activators such as AICAR (ATP-dependent) and A23187 (ATP-independent) could suppress cervical cancer cell growth in the presence or absence of LKB1, an upstream kinase of AMPK [10]. We also proposed mechanistic evidence showing that metformin, AICAR and A23187 suppress cervical cancer cell growth through reducing DVL3, a positive effector of Wnt/β-catenin signaling cascade which has been shown to be constitutively active during cervical cancer development [12]. Yet, it is still believed that there are other molecular mechanisms by which these pharmaceutical AMPK activators suppress cancer cell growth. The understanding of these mechanisms will assist in exploring better therapeutic regimes when using these drugs.

Forkhead Box M1 (FOXM1) is a member of the Forkhead Box transcription factors which is essential for cell proliferation and apoptosis in the development and function of many organs [13-17]. We previously reported that aberrant upregulation of FOXM1 is associated with the progression and development of human cervical squamous cell carcinoma (SCC) [18]. Biochemical and functional studies confirmed that FOXM1 is critically involved in cervical cancer cell growth through upregulating cyclin B1, cyclin D1 and cdc25B and downregulating p27 and p21 expressions. These findings suggest that FOXM1 plays a vital role in cervical cancer cell growth and oncogenesis.

In this study, we reported that the activated AMPK inhibits the cell growth by reducing FOXM1 expression in human cervical cancer cells upon treatments with hypoxia, glucose deprivation and pharmaceutical AMPK activators. We provided both biochemical and functional evidence to support our findings that the repression of FOXM1 expression of AMPK is dependent on the AKT/FOXO3a/FOXM1 signaling cascade.

## Methods

### Cell lines and reagents

Cervical cancer cell lines HeLa, CaSki, C33A and SiHa (American Type Culture Collection, Rockville, Md., USA) (cell line authentication was done by in-house STR DNA profiling analysis) were employed in this study. They were maintained in Dulbecco’s Modified Eagle Medium (DMEM) (Invitrogen, Carlsbad, CA) supplemented with 10% (v/v) fetal bovine serum (Gibco), 100 units/ml penicillin/streptomycin (Gibco) at 37°C in an incubator with humidified atmosphere of 5% CO_2_ and 95% air. AMPK activators AICAR, A23187 and metformin and AKT inhibitor LY294002 were obtained from Tocris Bioscience (Bristol, UK). FOXM1 inhibitor Thiostrepton was purchased from Calbiochem (La Jolla, CA, USA).

### Plasmids and cell transfection

To study the effects of enforced FOXM1 expression, the FOXM1c-expressing plasmid pcDNA3–FOXM1c was used because the c isoform has higher transactivating activity and is expressed dominantly in cells as well as tissues. Whereas, the pcDNA3 empty vector was used in mock transfections as control. Besides, the vector-based shRNA plasmid pTER–FOXM1 was used to knockdown endogenous FOXM1. All of these plasmids had been described previously [18]. As controls in knockdown assays, the p-super GFP and pcDNA3 vectors were used in mock transactions. To knockdown human FOXO3a, the TriFECTa RNAi Kit which contains three siRNAs targeting human FOXO3a was purchased from IDT (Integrated DNA Technologies, Inc., Iowa, USA). Cell transfection was carried out using LipofectAMINETM 2000 (Invitrogen) according to the manufacturer’s instructions. Expression patterns were analyzed by Western blotting. The parental vector pEGFP-C1 was used as empty vector control.

### Cell proliferation assay

Cell proliferation kit (XTT) (Roche, Basel, Switzerland) was used to measure cell viability according to the manufacturer’s protocol. Three independent experiments were performed in triplicates.

### RNA extraction and quantitative reverse transcriptase–PCR (Q-PCR)

According to the instruction of the manufacturer, total RNA was extracted using TRIzol reagent (Invitrogen). Complementary DNA (cDNA) was subsequently synthesized using a reverse transcription reagent kit (Applied Biosystems, Foster City). The expression level of *FOXM1* was then evaluated by q-PCR in an ABI PRISM™ 7500 system (Applied Biosystems) using Taqman® Gene expression Assays; human *FOXM1* (Assay ID: Hs00153543_m1). The human *18S rRNA* (Assay ID: Hs99999901_m1) was used as an internal control.

### Western blot analysis

Proteins in cell lysates were separated by 10% SDS-PAGE and transferred to polyvinylidene-difluoride (PVDF) membranes. The membranes were blotted with 5% skimmed milk and subsequently probed overnight at 4°C with primary antibodies specific for p-AMPKα, AMPKα, p-AKT, AKT, p-FOXO3a, FOXO3a (Cell Signaling, Beverly, MA, USA), FOXM1 (Santa Cruz Biotechnology, Inc., Santa Cruz, CA, USA) and β-actin (Sigma-Aldrich, St. Louis, MO, USA) and then incubated with horseradish peroxidase conjugated goat anti-rabbit or anti-mouse secondary antibody (Amersham, Uppsala, Sweden). Immunodetection was performed with enhanced chemiluminescent reagent solution (Amersham^TM^ ECL^TM^) and visualized using medical X-ray film.

### Data analysis

Student’s *t* test was applied to the data analysis. All data were expressed as mean ± SEM. *P*-value of less than 0.05 was considered as significant.

## Results

### Increased AMPK activity inhibits cervical cancer cell growth by suppressing FOXM1 expression

**Figure 1:**
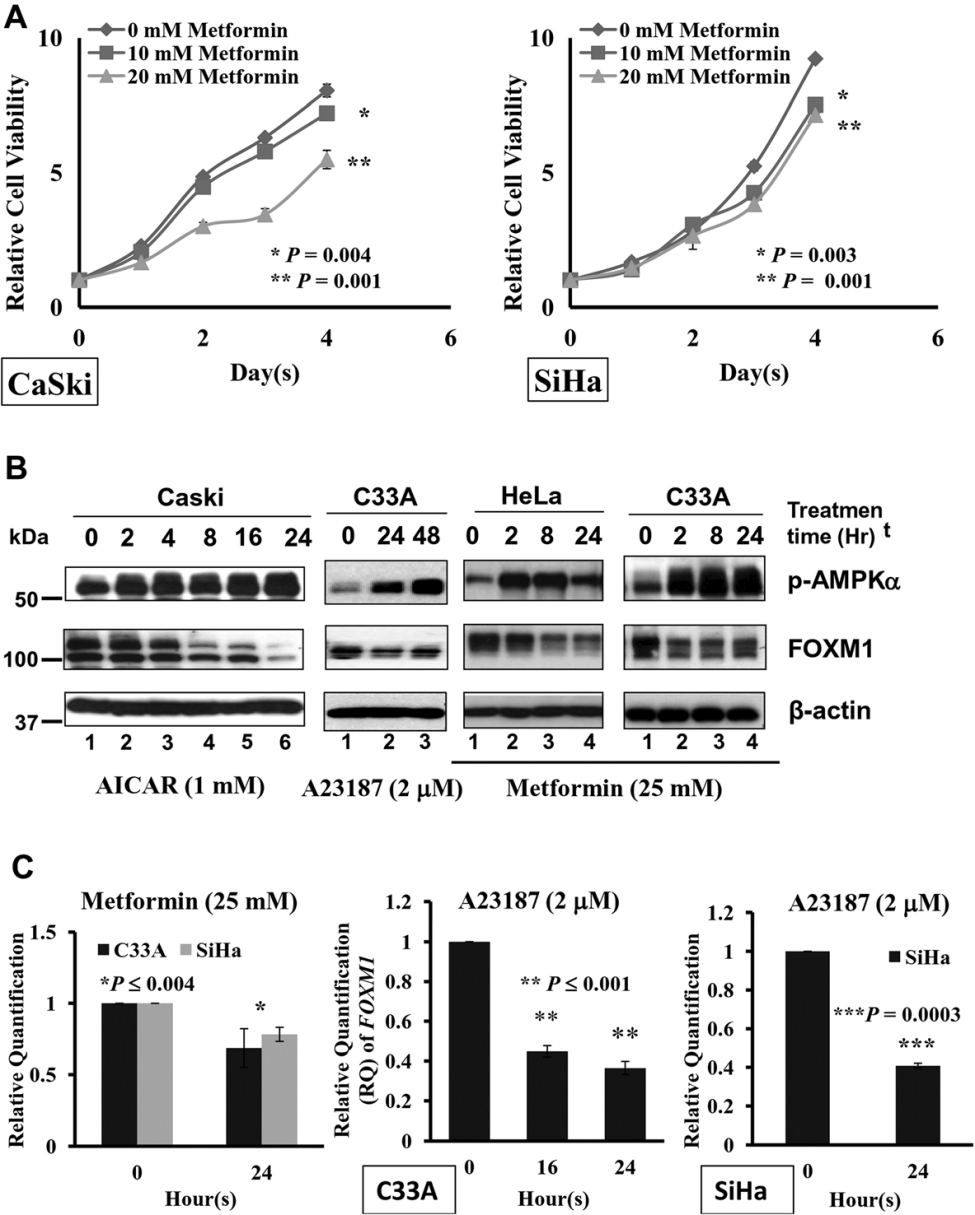
**Activation of AMPK by pharmaceutical AMPK activators suppresses expression of FOXM1. (A)** XTT cell proliferation assay showed that the growth rates of cervical cells; Caski and SiHa, were significantly reduced in a dose and time dependent manner upon the treatment of metformin (10 mM and 20 mM) when compared with the control medium. **(B)** Western blot analysis showed that the level of p-AMPKα was increased, while the level of FOXM1 was reduced in cervical cancer cells (Caski, C33A and Hela) when treated with AICAR (1 mM), A23187 (2 μM) and metformin (25 mM) for various time points. **(C)** RT-Q-PCR analysis demonstrated that the *FOXM1* mRNA level of C33A and SiHa cells was remarkably reduced when treated with metformin (25 mM) and A23187 (2 μM) from 16 hrs to 24 hrs. The *18S* gene was used as an internal control.

Consistent with our previous findings [12], this study also showed that the cell growth of cervical cancer cell lines such as Caski and SiHa was significantly inhibited by the AMPK activator metformin on a time and dose dependent manner (Figure 1A). Similarly, other AMPK activators such as AICAR and A23187 displayed remarkable inhibitory effect on cervical cancer cells (data not shown), confirming that activation of AMPK is able to reduce cervical cancer cell growth.

**Figure 2:**
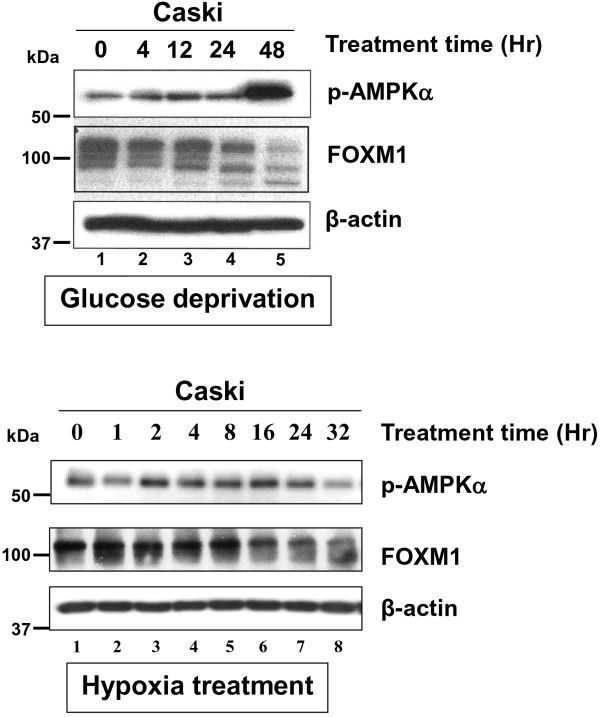
**Activation of AMPK by glucose deprivation and hypoxia inhibits cell growth in cervical cancer cells.** Caski cells treated by glucose deprivation and hypoxia at various time intervals showed an increased of p-AMPKα but a reduction of FOXM1.

As FOXM1 is a master regulator of cancer cell growth, it is of interest to examine whether increased AMPK activity has any functional impact on FOXM1 in cervical cancer oncogenesis. Upon treatment of AICAR (1 mM), A23187 (2 μM), metformin (25 mM), glucose deprivation and hypoxia on the cervical cancer cell lines Caski, C33A and HeLa, we found that FOXM1 expression was drastically decreased while AMPK activity [p-AMPK (Thr172)]was elevated concomitantly (Figure 1B and Figure 2). Interestingly, Q-PCR analysis also demonstrated that the mRNA level of FOXM1 in C33A and SiHa cells was remarkably reduced upon treatment of metformin (25 mM) and A23187 (2 μM) in a time dependent manner (Figure 1C). This finding indicates that AMPK activated by these pharmaceutical activators could suppress FOXM1 expression at both protein and mRNA levels.

### Activated AMPK represses FOXM1 expression through blocking the AKT/FOXO3a signaling pathway

**Figure 3:**
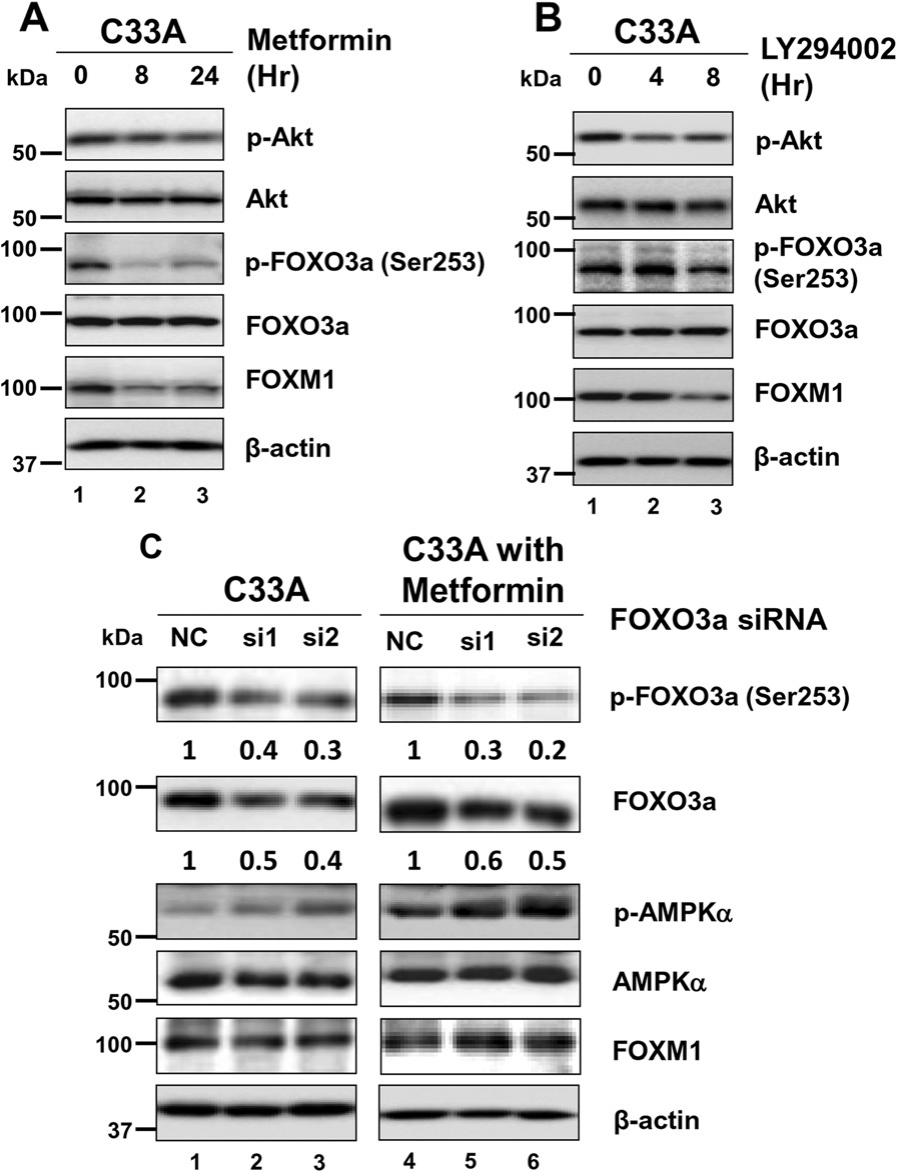
**Activation of AMPK represses FOXM1 expression through inhibiting the AKT/FOXO3a signaling pathway. (A)** Upon treatment of metformin (25 mM) in cervical cancer cells (C33A), the expression of FOXM1 diminished remarkably and the phosphorylation of AKT and AKT-specific phosphorylation of FOXO3a (Ser253) decreased concomitantly. **(B)** Co-treatment of PI3K/AKT inhibitor, LY294002 (10 μM), inhibited p-AKT and concomitantly reduced the levels of FOXO3a (Ser253) and FOXM1 in C33A cells. **(C)** Depletion of endogenous FOXO3a in C33A cells using siRNAs showed that FOXO3a expression is required for the suppression of FOXM1 by metformin (25 mM) treatment.

FOXO3a is well known to be one of the FOXO transcription factors that functions downstream of PI3K-PTEN-AKT (PKB) signaling in modulating cell growth [19]. Along the signaling cascade, FOXO3a is a negative regulator of FOXM1 expression [20]. To investigate whether the suppressive effect of AMPK on FOXM1 is mediated via FOXO3a, we first examined the intensity of FOXO3a dephosphorylation by metformin. Upon treatment of metformin (25 mM) on the cervical cancer cell line C33A, not only FOXM1 expression diminished remarkably but also the phosphorylation of AKT and the AKT-specific phosphorylation of FOXO3a (Ser253) (Figure 3A). We then examined whether the PI3K/AKT inhibitor LY294002 could reduce FOXM1 expression in cervical cancer cells. As expected, C33A cells exhibited decreased intensity of AKT-specific phosphorylation of FOXO3a (Ser253) as well as FOXM1 expression upon the treatment of LY294002 (10 μM) (Figure 3B). To further assess whether FOXO3a is primarily involved in the reduction of FOXM1 induced by AMPK activation but not an off-target pharmaceutical effect, siRNA-based knockdown of FOXO3a in C33A cells was carried out. Western blot analysis revealed that cervical cancer cells with depletion of endogenous FOXO3a did not show altered FOXM1 expression even when AMPK was activated subsequently by metformin (25 mM) (Figure 3C). Taken together, our data support that inhibition of FOXM1 by AMPK activation is attributed to the repression on AKT and its downstream, AKT-specific phosphorylation of FOXO3a (Ser253) in cervical cancer cells.

### Ectopic expression of FOXM1 rescues AMPK-mediated cell growth inhibition

**Figure 4:**
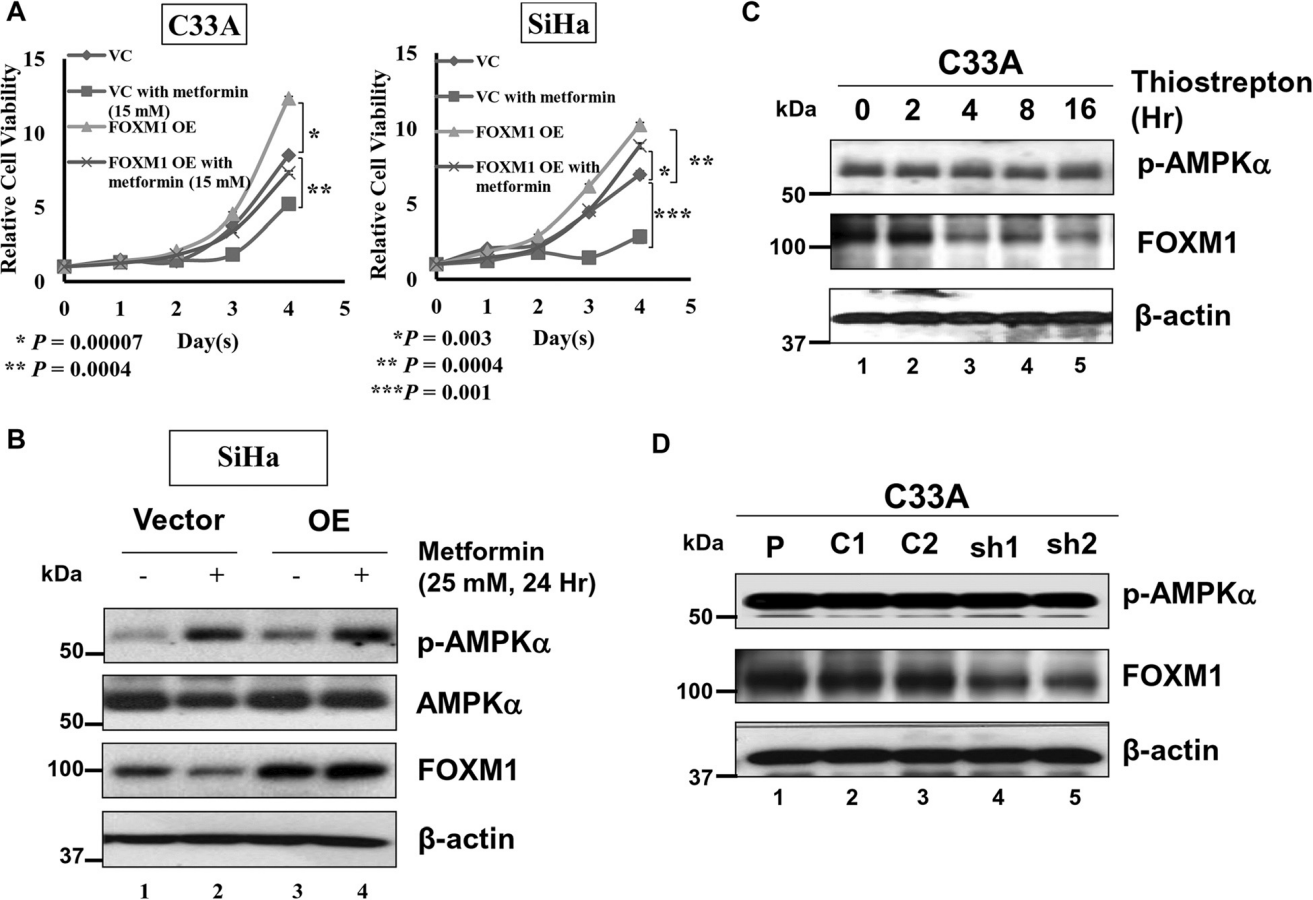
**Ectopic expression of FOXM1c could rescue AMPK mediated cell growth inhibition in cervical cancer cells. (A)** XTT cell proliferation assay showed that the growth rates of C33A and SiHa cells with ectopic expression of FOXM1c (OE) were significantly increased as compared with empty vector control (VC). C33A and SiHa cells with ectopic expression of FOXM1c showed a less inhibitory effect on cell growth rate as compared with the vector controls upon treatment of metformin (20 mM). **(B)** Western blot analysis showed that the level of FOXM1 (including exogenous FOXM1c) could not be reduced in SiHa cells when treated with metformin (25 mM) for 24 hrs. **(C)** The level of p-AMPKα remained constant when C33A cells were treated with FOXM1 specific inhibitor, thiostrepton (5 μM). **(D)** Depletion of the endogenous FOXM1 by shRNA knockdown approach could not affect the level of p-AMPKα. Both sh1 and sh2 represent two independent pTER–FOXM1 transfections, while C1 and C2 represent the vector controls.

Given that activation of AMPK leads to growth inhibition of cervical cancer cells through reduction of both the mRNA and protein levels of FOXM1, we sought to determine whether enforced expression of exogenous FOXM1 could counteract the AMPK-induced suppressive effect. C33A and SiHa cells were transiently transfected with FOXM1c-expressing plasmid and treated with metformin (20 mM). Consistent with our previous findings [18], XTT cell proliferation analysis showed that enforced expression of FOXM1c significantly promoted cell proliferation in both C33A (*P* = 0.00007) and SiHa (*P* = 0.0004) cells as compared with the vector control (Figure 4A). Importantly, C33A and SiHa cells with ectopic expression of FOXM1c could significantly reduce the effect of AMPK-mediated cell proliferation inhibition as compared with their vector controls upon treatment of metformin (20 mM) (Figure 4A). Such counteracting effect of ectopic FOXM1c was particularly evident in SiHa cells (Figure 4A). Indeed, Western blot analysis confirmed that there was no reduction in the expression of FOXM1 in FOXM1c-transfected SiHa cells upon treatment of metformin (25 mM) for 24 hrs (Figure 4B). Collectively, these findings confirm that activation of AMPK by hypoxia and glucose deprivation, as well as pharmacological AMPK activators inhibits cervical cancer cell growth, and this effect is dependent on the endogenous expression level of FOXM1.

### FOXM1 acts as an AMPK downstream effector

**Figure 5:**
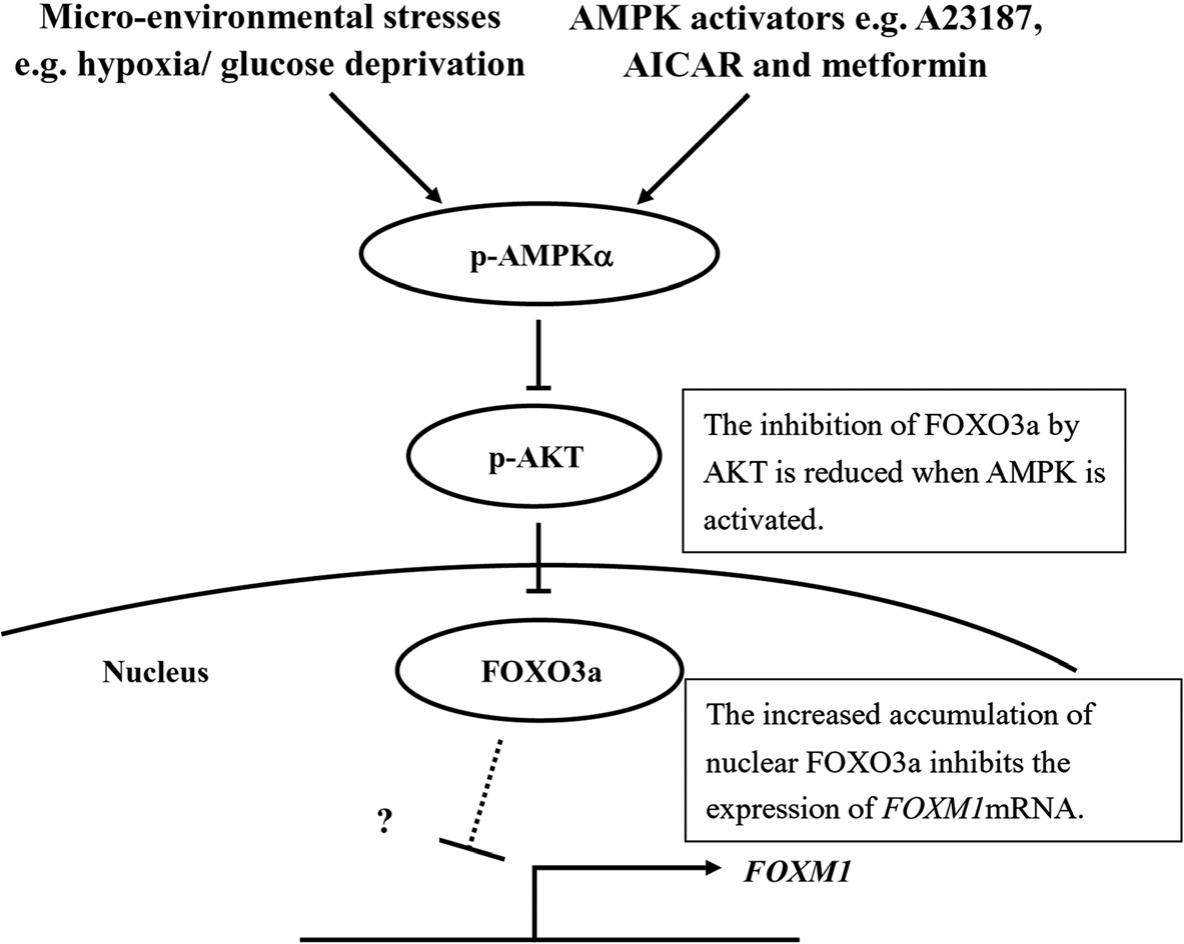
A schematic diagram to illustrate AMPK/AKT/FOXO3a/FOXM1 as the proposed molecular mechanism by which activated AMPK down-regulates FOXM1 expression.

Previous experiments have demonstrated that reduction of FOXM1 is a common scenario when AMPK is activated in cervical cancer cells. As FOXM1 is a key transcription factor, we sought to determine whether alteration of FOXM1 levels causes a feedback control on AMPK activity. To this end, we treated C33A cells with the FOXM1 specific inhibitor thiostrepton to suppress FOXM1 expression. Upon treatment of thiostrepton (5 μM), FOXM1 expression was significantly suppressed, whereas the expression of p-AMPKα (Thr172) was unchanged (Figure 4C). Similarly, knockdown of endogenous FOXM1 using shRNAs did not reveal any discernible change on the expression level of p-AMPKα (Thr172) in C33A cells (Figure 4D). Taken together, p-AMPKα activity per se is not altered by FOXM1 suppression induced by thiostrepton treatment or shRNA knockdown, implying that AMPK is acting upstream of FOXM1 and there is no feedback loop. Our analysis strongly supports that increased AMPK activity down-regulates FOXM1 through the AKT/FOXO3a/FOXM1 signaling cascade (Figure 5).

## Discussion

Recent studies have suggested that targeting cancer cell metabolism is an alternative therapeutic approach in cancer treatment. AMPK is a pivotal energy sensor governing normal and cancer cell metabolism. Our previous research has shown that pharmaceutical AMPK activators are able to repress cervical cancer cell growth through targeting DVL3 in the Wnt/β-catenin signaling pathway [12]. In this study, we report another molecular mechanism by which AMPK can retard cervical cancer cell growth: inhibition of FOXM1 function via AMPK/AKT/FOXO3a signaling. We demonstrated that AMPK activated by either micro-environmental stresses or pharmaceutical AMPK activators could reduce FOXM1 expression through blocking the AKT/FOXO3a signaling pathway, that in turn impaired cervical cancer cell growth.

The Forkhead box transcription factor FOXM1 regulates a number of key cell cycle regulators that control the G_1_ to S and the G_2_ to M transitions [21-26]. Accumulating evidences have shown that the upregulation of FOXM1 is often involved in the development of various human cancers [27-32]. We previously reported that there is a progressive increase in FOXM1 level in the progression of human cervical cancer [18]. The inhibition of FOXM1 by genetic or pharmaceutical approach significantly impairs tumor growth of this cancer *in vitro* and *in vivo*[18,25,27,33], suggesting that targeting FOXM1 is an appealing and potential approach for anticancer therapeutics. On the other hand, we and others have proved that activation of AMPK is able to inhibit the growth of various human cancers including cervical cancer [10,34-36]. The pharmacological activation of AMPK using AICAR or metformin has been shown to inhibit cell growth and induce cell apoptosis of a wide spectrum of cancer cells through modulation of p53 [37], p27 [38,39], or p21 [18,40], or DVL3 in Wnt/β-catenin signaling in cervical cancer [12]. Herein, we demonstrated that the activation of AMPK by various AMPK activators or hypoxia and glucose deprivation stresses induces a remarkable reduction of FOXM1 which in turn leads to a remarkable decrease of cervical cancer cell growth in both HPV positive (Caski, Hela and SiHa) and HPV negative (C33A) cell lines. On the other hand, ectopic expression of FOXM1c could counteract the suppressive effect of activated AMPK. These findings indicate that FOXM1 is a key oncogenic factor associated with cervical cancer cell growth, while activated AMPK inhibits cervical cancer cell growth through downregulation of endogenous FOXM1.

In fact, we demonstrated that reduction of FOXM1 occurred at both the mRNA but and protein levels in cervical cancer cells. This is suggestive of a transcriptional suppression of FOXM1 by its upstream effectors. Previous studies have reported that FOXM1 is transcriptionally suppressed by FOXO3a, which is a critical downstream effector of the PI3K/AKT/FOXO signaling pathway [19,20]. For example, it has been reported that FOXO3a represses estrogen receptors α (ERα) activity in breast cancer cells through an alternative mechanism by which FOXO3a interacts and downregulates the expression of FOXM1 [29,41]. These evidences imply that the reduction of FOXM1 at both the mRNA and protein levels is due to the presence of FOXO3a in cervical cancer cells. In fact, our data using siRNA-mediated FOXO3a knockdown showed that FOXO3a expression is required for FOXM1 reduction upon activation of AMPK.

How do activated AMPK leads to FOXO3a accumulating in the nucleus and blocking the transcription of *FOXM1* mRNA? FOXO3a, which belongs to the class O of Forkhead/winged helix box (FOXO) transcription factors, is a key tumor suppressor involved in different cellular processes [42]. FOXO3a is modified by phosphorylation, acetylation and ubiquitination, which in turn affect its nuclear/cytoplasm shuttling, transcriptional activity and stability [43-46]. It is known that the PI3K/AKT signaling is the main regulatory pathway of FOXO3a [44,47,48]. When PI3K/AKT signaling is activated, FOXO3a is not only inactivated and phosphorylated at Thr32, Ser253 and Ser315 residues, but is also exported out from the nucleus to the cytoplasm where it is ubiquitinated and subjected to proteasome-dependent degradation [43,48]. Therefore, nuclear FOXO3a functions as transcriptional regulator, whereas cytoplasmic FOXO3a is considered inactive [46]. On the other hand, AKT is a signaling kinase known to be inactivated by activated AMPK [49,50]. In our study, treatment of either AMPK activator (metformin) or PI3K/AKT inhibitor (LY294002) showed significant inhibition of p-AKT and a remarkable reduction of p-FOXO3a (Ser253), an AKT-specific phosphorylation site, suggesting that suppression of FOXO3a is reduced. As a result, FOXO3a will be more nuclear-localized and activated to inhibit *FOXM1* mRNA expression in cervical cancer cells.

Aforementioned, AMPK activation can commonly inhibit FOXM1 expression in cervical cancer cells. However, whether there exists a feedback loop on the activity of AMPK is still unknown. To test this notion, cervical cancer cells were treated with the FOXM1 inhibitor thiostrepton to investigate the effect on AMPK activation. Results showed that treatment of thiostrepton only reduced expression of FOXM1 but not the activity of AMPK. In addition, depletion of endogenous FOXM1 using shRNAs gave similar findings as the treatment of thiostrepton, implying that FOXM1 is acting downstream of AMPK without any feedback regulation.

## Conclusion

In summary, our findings here prove that activation of AMPK frequently inhibits cervical cancer cells. More importantly, we demonstrated that activated AMPK reduces FOXM1 by counteracting the AKT/FOXO3a/FOXM1 signaling axis. Our findings shed light on the application of AMPK activators in the treatment of human cervical cancer.

## Competing interests

The authors declare that they have no competing interests.

## Authors’ contributions

MY and DC designed research; MY, DC and VL performed the experiments; DC, VL and KY contributed new reagents-analytic tools; MY, DC and HN analyzed and interpreted data; MY and DC wrote the manuscript. All authors were involved in editing the manuscript and had final approval of the submitted and published versions.

## Pre-publication history

The pre-publication history for this paper can be accessed here:

http://www.biomedcentral.com/1471-2407/13/327/prepub
